# Effect of low-intensity pulsed ultrasound on the duration of mandibular anterior crowding correction and pain perception in adults undergoing orthodontic treatment: a single-blinded randomized control trial

**DOI:** 10.1590/2177-6709.30.1.e2524158.oar

**Published:** 2025-04-07

**Authors:** Saavi SOROUT, Poornima JNANESHWAR, Ravi KANNAN

**Affiliations:** 1 SRM Dental College, Department of Orthodontics (Ramapuram, Chennai, India).

**Keywords:** Low-Intensity Pulsed Ultrasound, Alignment and leveling, Orthodontic tooth movement, Ultrassom pulsado de baixa intensidade, Alinhamento e nivelamento, Movimentação dentária ortodôntica

## Abstract

**Objectives:**

This single-blinded randomized control trial aimed to assess the effect of Low-Intensity Pulsed Ultrasound (LIPUS) on the duration of alignment and leveling stage, and to assess patients’ pain and discomfort following the procedure.

**Material and methods:**

30 participants (18-35 years) were recruited, of whom 22 were selected with Little’s Irregularity Index of 1-6mm following a non-extraction protocol, and were randomly allocated into two groups using a lottery method. The test group underwent the LIPUS application for 20 minutes on days 0, 3, 5, 7, 15, and every 15 days until completion of alignment and leveling. The control group was subjected to a placebo protocol. Stage models were fabricated every 21st day and the duration taken for completion of alignment was recorded. The patient’s satisfaction scale was documented using a questionnaire on a Visual Analog Scale.

**Results:**

A statistically significant reduction was found in treatment time for alignment and leveling. A 22% reduction in total duration for alignment and leveling was observed (p=0.027). No appreciable difference in pain and discomfort was observed between the groups.

**Conclusion:**

The application of LIPUS for 20 minutes on days 0, 3, 5, 7, 15, and every 15 days until alignment completion showed a significant reduction in the duration taken for alignment and leveling of teeth.

## RESUMO

**Objetivos:**

Este ensaio clínico randomizado, cego e controlado teve como objetivo avaliar o efeito do ultrassom pulsado de baixa intensidade (LIPUS) na duração do estágio de alinhamento e nivelamento, e avaliar a dor e o desconforto dos pacientes após o procedimento.

**Material e Métodos:**

Foram recrutados 30 participantes (18-35 anos), dos quais 22 foram selecionados, com Índice de Irregularidade de Little de 1-6mm, seguindo um protocolo de não extração, e foram alocados aleatoriamente em dois grupos por meio de sorteio. O grupo teste foi submetido à aplicação do LIPUS por 20 minutos, nos dias 0, 3, 5, 7, 15 e a cada 15 dias até a finalização do alinhamento e nivelamento. O grupo controle foi submetido a um protocolo placebo. Modelos de gesso foram confeccionados a cada 21 dias, e o tempo necessário para a conclusão do alinhamento foi registrado. O nível de satisfação do paciente foi documentado por meio de um questionário com Escala Visual Analógica.

**Resultados:**

Foi encontrada redução estatisticamente significativa no tempo de tratamento para o alinhamento e nivelamento. Foi observada redução de 22% no tempo total de alinhamento e nivelamento (p=0,027). Nenhuma diferença significativa na dor e no desconforto foi observada entre os grupos.

**Conclusão:**

A aplicação do LIPUS por 20 minutos nos dias 0, 3, 5, 7, 15 e a cada 15 dias até a finalização do alinhamento resultou em redução significativa no tempo de alinhamento e nivelamento dos dentes.

## INTRODUCTION

An effective orthodontic treatment is a combination of structural balance, functional efficiency, and aesthetic harmony. A fourth factor, “time”, warrants equal consideration in the present era. There is an increasing demand for a reduction in orthodontic treatment duration amongst the population.

Treatment duration in orthodontics varies with the severity of malocclusion; with a minimum of one year for simple malocclusion to more than two and half years in addressing skeletal discrepancy of jaws. Patients undergoing orthodontic treatment might not be comfortable with the prolonged treatment time.^1^

The periodontal structures of adult patients are influenced by many factors, like bone loss leading to a shift in the center of resistance (CRes) of teeth, which requires modified biomechanics. Excessive calcification leads to sclerosis of bone that might increase the resistance to orthodontic tooth movement. Aging dentition becomes susceptible to root resorption on application of orthodontic force.^1^ The aesthetic concern of adult patients forces them to demand a shorter treatment time. This has led to an increase in research in the field of accelerated orthodontics.

Innumerable techniques have been introduced to accelerate orthodontic tooth movement (OTM). These include surgical- Periodontally Accelerated Osteogenic Orthodontics (PAOO), Piezocision and Micro-osteoperforations (MOP), physical- vibratory stimulus, Low-Level Laser Therapy (LLLT), and Low-Intensity Pulsed Ultrasound (LIPUS), and chemical agents and 3 mediators- PgE1, Misoprostol, 1,25-Dihydroxycholecalciferol, Parathyroid hormones, IV immunoglobulins and platelet-rich fibrin.^2^ ^-^ ^9^

Ultrasound therapy was introduced in medicine for two purposes, diagnostic and therapeutic. Diagnostic frequency ranges from 1.0 to 20.0 MHz, and therapeutic ranges from 1 to 3.0 MHz. Intensities may vary from low (<3W/cm^2^) to high (>3W/cm^2^).^10^ In 1950, Buchatala^11^ suggested the stimulatory effect of ultrasound on bone osteogenesis. Since then, ultrasonic waves have been used to treat bone fractures. High-intensity ultrasound waves were used to activate bone osteogenesis until the introduction of LIPUS by Duarte.^12^

The healing effects of ultrasound in dentistry have proven effective in TMJ disorders, myofascial pain, and distraction osteogenesis.^13^ ^,^ ^14^ LIPUS has been used to promote periodontal ligament regeneration, restoring damaged dental roots and decreasing root resorption.^15^ LIPUS is most commonly used for therapeutic purposes in medicine and dentistry with an average intensity of 30mW/cm^2^ and a frequency of 1.5 MHz.^16^ It was introduced in orthodontics to expedite tooth movement by the pioneering research of El-Bialy et al.^15^ ^,^ ^17^, who concluded that there was a significant surge in the cell count in the sub-odontoblastic layer of rat teeth and periodontal ligament, with a dose-dependent effect after LIPUS application for 5 and 10 minutes/day for 5 days.

Liu et al.^18^ and Raza et al.^19^ revealed the healing property of LIPUS on orthodontically induced root resorption (OIRR) through a decrease in expression of osteoclasts. Xue et al.^20^ proved that LIPUS increased OTM by elevating the activity of HCG/RunX2/BMP-2 pathway and RANKL. Numerous authors have performed research to test the efficacy of LIPUS on OTM and found that there was an increase in the rate of tooth movement, thus proving that LIPUS can be utilized as an aid for accelerating tooth movement.^15^ ^,^ ^21^ ^,^ ^22^

There is sufficient research on the efficiency of LIPUS on the rate of canine retraction and *en-masse* retraction of anterior teeth. ^15^ ^,^ ^21^ ^,^ ^22^ Literature evidence evaluating the effect of LIPUS on the alignment of mandibular incisors is scarce. Hence the present study aimed to evaluate the duration taken for alignment of mandibular anterior teeth after the application of LIPUS in human subjects undergoing fixed orthodontic treatment, and compare it with control subjects undergoing fixed orthodontic treatment. The second objective was to assess the pain and discomfort experienced by patients following the LIPUS application, and compare them with control subjects. The null hypothesis stated was that there would be no difference in duration for alignment and leveling between the LIPUS application and the control group.

## METHODOLOGY

Trial design: The present study was a single-blinded parallel randomized control trial, with an allocation ratio of 1:1.

Sample size calculation: The sample size determination for this single-blinded randomized control trial was done using G*Power software, for a power of 80%, with an alpha error of 0.05 and an effect size of 0.891. The total sample size was calculated as 22. 

Participants: Approval for the study was obtained from the Institutional Review Board and Clinical Trial Registry. Thirty patients were recruited from the subjects reporting in the Department of Orthodontics in SRM Dental College (Ramapuram, Chennai, India), keeping in mind the sample size of 22 and further attrition that might occur throughout the research. Twenty-two subjects were selected from the 30 recruited. The eligibility criteria for participant selection were mild to moderate crowding in the mandibular anterior region.

Inclusion and exclusion criteria - Subjects in the age group of 18 to 35 years regardless of gender, presence of full dentition with permanent teeth, with or without third molars, subjects requiring non-extraction orthodontic treatment protocol, presence of mild to moderate lower anterior crowding (discrepancy of 1-6mm) assessed by Little’s Irregularity Index, good oral hygiene and sufficient thickness of attached gingiva were included in the study. Subjects with smoking habits, probing depth of the gingival sulcus exceeding 3 mm, and chronic periodontal disease leading to bone loss were excluded from the study.

Randomization: The subjects were randomly allocated into experimental and control groups by simple lottery method. 

**Figure 1:**
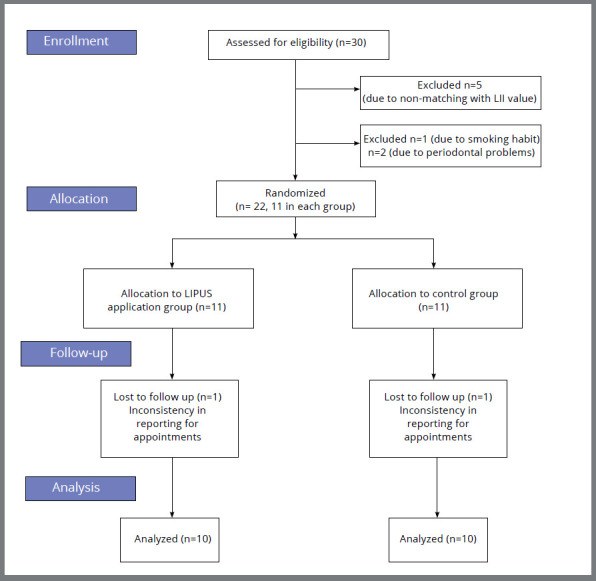
CONSORT flow diagram.

**Figure 2:**
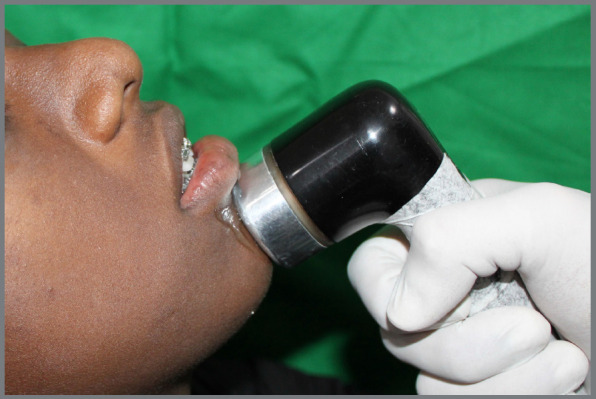
Extraoral LIPUS application on human subject.

Intervention: Orthodontic treatment was carried out using ORMCO Mini Diamond 0.022 x 0.028-in MBT brackets. Non-extraction treatment protocol for all 22 subjects was confirmed after model and cephalometric analysis. Little’s Irregularity Index (LII) was calculated using a caliper device (SKADIOO electronic caliper, Perfect Sales India) with 0.1-mm resolution and 0.2-mm accuracy to assess the discrepancy, which was found to be between 1 and 6 mm. Figure 1 depicts the flowchart explaining participant enrollment and reasons for the exclusion of some subjects (n=8) from the study. Consent was obtained after the treatment protocol for this research study was explained to the subjects. After bonding and initial archwire placement, experimental group patients were subjected to the LIPUS (Prestigious Global Enterprise, UMS) application starting from the day of bonding (day 0) and on days 3, 5, 7, 15, and every 15th day until the completion of alignment and leveling. The protocol was first described by Maurya et al.^21^ LIPUS application was done extraorally on the chin, coinciding with the position of lower anterior teeth, for 20 minutes at an intensity of 0.3W/cm^2^, using propylene glycol gel to conduct the waves (Fig 2).

Blinding: The control group had a LIPUS device placed extraorally on the chin without switching it on, to maintain blinding of subjects.

**Figure 3:**
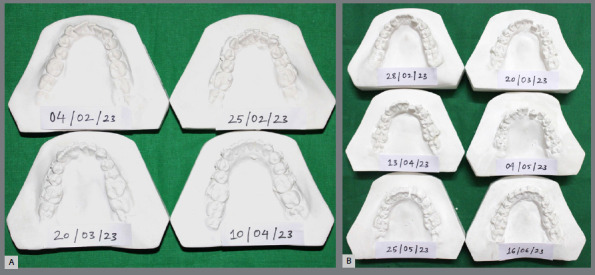
Stage models for the LIPUS application group (A) and for the control group (B).

Outcome measures: Alginate impression and intraoral photograph of the mandibular arch were taken every 21^st^ day until the passive engagement of 0.019 x 0.025-in stainless steel archwire in the mandibular arch (Fig. 3). LII was measured at every interval, to estimate the progress of alignment and leveling. The mean duration (days) for the alignment of mandibular anterior teeth was recorded for all the subjects in the experimental and control groups. The reduction in the percentage of treatment duration was calculated using the following formula:^15^

$$\begin{array}{l} overall\;reduction\;in\;alignment\;duration\%\\ =\frac{\left(\;Mean\;duration\;of\;alignment\;in\;control\;-\;mean\;duration\;of\;alignment\;in\;LIPUS\;group\;\right)}{\;mean\;duration\;of\;alignment\;in\;control\;}\times 100 \end{array}$$

Pain and discomfort in the experimental and control groups subjects were assessed using a questionnaire. The score was recorded using a Visual Analog Scale (VAS), which followed a grading from 0-10 where: 0 denoted no pain/discomfort and 10 denoted excruciating/ unbearable pain. 

## STATISTICAL ANALYSIS

The mean duration for alignment and leveling was calculated for the experimental and control groups. The data were analyzed using IBM SPSS (IBM Corp. Released 2011. IBM SPSS Statistics for Windows, version 20.0. Armonk, NY: IBM Corp). The normality of data was tested using the Kolmogorov-Smirnov and Shapiro-Wilk tests.

As the data were normally distributed, an unpaired t-test was conducted to compare the duration taken for alignment between the groups.

The data from the questionnaire part of the study assessing the pain and discomfort of the patient was not normally distributed, hence Mann-Whitney U test was conducted. 

## RESULTS

Participants: Participant recruitment for the study was started on 1^st^ November 2022 and completed on 1^st^ October 2023. The data of 2 patients, one from each group, was removed due to the inconsistency in appointment reporting (Fig. 1).

Baseline data: Demographic analysis was performed by comparing the mean age of subjects in both groups. When subjected to an Unpaired t-test, it was found that there was no significant difference in the age of subjects in both groups (Table 1). 

**Table 1:** Demographic data comparing the age of samples in the two groups.

Age (years)						
	Mean	SD	SEM	95% confidence Interval of the difference		p-value
				Lower	Upper	
Experimental	22.72	5.34	1.41	-5.39101	3.57283	0.677
Control	21.81	4.70	1.61			

SEM = standard error of mean.

Primary outcome: The mean number of days taken for alignment in the experimental and control groups were 120.0±24.23 days and 154.10±37.53 days, respectively (Table 2). The statistical test found a significant reduction in duration taken for alignment and leveling in the LIPUS group (p= 0.027). Thus, the null hypothesis was rejected. A clinically significant reduction in duration for alignment was observed in the experimental group (22%) (Table 2). 

**Table 2:** Descriptive statistics, unpaired t-test comparison, and percentage reduction in duration taken for alignment and leveling in experimental and control.

Total duration (days) (n=10)							
	Mean	SD	SEM	95% confidence Interval of the difference		p-value	% reduction
				Lower	Upper		
Experimental	120.20	24.23	7.66	-63.587	-4.212	0.027	22%
Control	154.10	37.53	11.87				

SEM = standard error of mean.

Secondary outcome: Patient satisfaction with appliance usage was checked using a questionnaire, and the patients were instructed to record the level of pain and discomfort on a Visual Analog Scale (VAS). Results revealed no statistically significant difference in the pain and discomfort between the groups (Table 3).

**Table 3:** Descriptive statistics, Mann-Whitney U test for comparing the pain and discomfort levels in experimental and control group.

Question		LIPUS group	Control group	Mann-Whitney U	P value
Pain score					
1	Pain after the procedure	0.7273	1.4545	45.000	0.252
2	Pain 24 hrs after the procedure	1.1818	2.0000	48.000	0.379
3	Pain 48 hrs after the procedure	1.1818	2.0909	44.500	0.268
4	Pain during eating and locomotory movement	0.5455	0.0000	55.000	0.317
5	Pain during sleeping at night	0.7273	1.3636	48.000	0.378
Discomfort score					
1	Discomfort during the procedure	0.0000	0.0000	60.500	1.000
2	Discomfort at the site of the device application	0.0000	0.0000	60.500	1.000

## DISCUSSION

In the present study, LIPUS was applied to human subjects to assess its effect on the duration of alignment and leveling of teeth. There was a 22% reduction in duration taken for alignment and leveling in the LIPUS application group (Table 2). The mean of total duration for alignment and leveling for the experimental group was reduced when compared to the control group (120.0 ± 24.23 days and 154.10 ± 37.53 days) and the result was significant (p=0.027) (Table 2). The null hypothesis was thus rejected.

El-Bialy et al.^15^ assessed the amount of orthodontic tooth movement (OTM) per week in subjects requiring canine retraction following LIPUS application, and concluded that OTM was faster in the LIPUS group. Qamruddin et al.^22^ evaluated the potency of LIPUS application at 3-week intervals on the rate of canine retraction, and found that there was no acceleration of tooth movement with LIPUS. A probable reason for the insignificant results in their study could be due to the reduced frequency of LIPUS application. Maurya et al.^21^ studied the rate of canine retraction with LLLT and LIPUS application, compared to control groups. There was a distinct reduction in the duration of space closure in the LIPUS group.

Lo Giudice et al.^23^ conducted a study to examine the efficiency of photobiomodulation (PBM) using a device of 450 to 835 nm wavelength, and concluded that the median treatment time was remarkably shorter in the PBM group, in comparison to the control. The current study results are congruent with those of Nahas et al.^24^ and Ghafar et al.^25^, who evaluated the effect of LLLT on the alignment of the mandibular anterior teeth and concluded that the alignment duration for the test group was smaller than in the control.

In the present study, LIPUS was used as an accelerating method and its effect on the duration of alignment of mandibular anterior teeth was assessed. Alsino et al.^2^ evaluated the efficacy of Periodontally Accelerated Osteogenic Orthodontics (PAOO) in the correction of lower anterior crowding, and reported an acceleration in the rate of alignment and leveling by 40%. Al-Ibrahim et al.^3^, evaluated the effect of self-ligating brackets with flapless piezocision on severely crowded upper anterior teeth, and concluded that piezocision led to a faster reduction in Little’s Irregularity Index. Gibreal et al.^4^ reported a reduction of 59% in the overall alignment time of lower anterior teeth in subjects treated with piezocision-based flapless corticotomy. Al-Attar et al.^26^ concluded that the duration for mandibular teeth alignment using micro-osteoperforations (MOPs) was significantly shorter than the control group. 

LIPUS has many clinical advantages, including the fact that it is a biological stimulus, easy to use, and non-invasive, and has been widely used in clinical medicine.^17^ It is a portable, compact device and can be used in daily practice for accelerating tooth movement.

The current study examined pain and discomfort with and without LIPUS application, using a questionnaire. The statistical analysis showed no appreciable difference in the levels of pain and discomfort between the groups (Table 3). Similar results were obtained by Qamruddin et al.^22^, who evaluated the pain intensity and found no significant difference in pain between the LIPUS application and the control group. 

Al-Hanbali et al.^27^ evaluated the effect of LIPUS and LLLT on pain perception after installing orthodontic separators, and concluded a significant reduction amongst the groups with LIPUS and LLLT application.

Other acceleration methods have been evaluated regarding their efficacy on pain perception. Alsino et al.^28^ concluded high levels of pain and discomfort on the first day in patients undergoing PAOO, which decreased significantly over the following appointments. Al-Ibrahim et al.^29^ reported a statistical increase in pain and discomfort in subjects who underwent piezocision therapy. Gibreal et al.^30^ and Sirri et al.^31^ reported no significant difference in pain perception between corticotomy and control groups during the alignment of lower anterior teeth. 

### Limitations of this study

Documentation on the outcome of LIPUS application on OTM is very limited and has been evaluated in extraction protocol. The results of the current study implied that there was an appreciable reduction in the duration of alignment and leveling of mandibular anterior teeth in subjects who had extraoral LIPUS application on days 0, 3, 5, 7, and 15, and every 15th day until the passive engagement of 0.019 x 0.025-in stainless steel wire in 0.022 x 0.028-in MBT slot, when compared to the control group. Thus, the null hypothesis was rejected. 

### Future scope

The mechanism of action of LIPUS on OTM was not studied. This remains a limitation of the present study, in addition to the small sample size. Future research should focus on different protocols of LIPUS application and their effect on OTM, to assess the one providing the best results. The molecular basis of the action of LIPUS on orthodontic tooth movement should be studied to get clarity on the effect of LIPUS on orthodontic tooth movement.

## CONCLUSIONS

The duration taken for alignment and leveling in human subjects undergoing fixed orthodontic treatment subjected to LIPUS application was less than that of the control group, and the difference was statistically significant (p=0.027).

No statistically significant difference in patient’s perception of pain and discomfort was observed between the groups.
